# 4199 A pilot randomized controlled trial of precision care for smoking cessation in the Southern Community Cohort Study

**DOI:** 10.1017/cts.2020.323

**Published:** 2020-07-29

**Authors:** Nicole Senft, Maureen Sanderson, Rebecca Selove, William J. Blot, Rachel F. Tyndale, Quiyin Cai, Karen Gilliam, Suman Kundu, Hilary A. Tindle

**Affiliations:** 1 Vanderbilt University Medical Center; 2 Meharry Medical College; 3 Tennessee State University; 4 University of Toronto

## Abstract

OBJECTIVES/GOALS: Precision care may engage smokers and providers in treatment but is understudied in the community. We piloted guideline-based care (GBC) alone or with Respiragene, a lung cancer polygenic risk score (PRS, 1-10), or metabolism-informed choice of medication using the nicotine metabolite ratio (NMR). METHODS/STUDY POPULATION: Daily smokers (n = 58) with stored biospecimens in the Southern Community Cohort Study were randomized 1:1:1 to GBC, PRS, or NMR, counseled to quit smoking, and co-selected FDA-approved cessation medication (nicotine replacement, varenicline) with a tobacco counselor. In PRS, precision motivational counseling was guided by PRS (i.e., lung cancer risk 10-40-fold that of never-smokers). In NMR, precision medication recommendations consisted of varenicline for faster metabolizers (NMR≥0.31) and nicotine replacement for slow metabolizers (NMR<0.31). Feasibility was defined as achieving at least 50% provider engagement (med prescription) and at least 50% patient engagement (self-reported med use). RESULTS/ANTICIPATED RESULTS: Participants were median age 59, 72% female, 81% Black, 60% with incomes <$15,000; median cigarettes/day was 15 (IQR 8-20) and 52% reported time-to-first cigarette <5 minutes, illustrating moderate nicotine dependence. Providers confirmed medication prescriptions for 40% of patients (32% GBC, 50% PRS, 37% NMR) and 83% of patients reported using medication (prescribed or unprescribed) during the study (90% GBC, 80% PRS, 79% NMR). At 6-month follow-up, 27% (n = 15) reported cessation (39% GBC, 16% PRS, 26% NMR). Among persistent smokers, 46% reported smoking at least 50% fewer cigarettes/day compared to baseline (45% GBC, 38% PRS, 57% NMR). Small sample size precluded statistical comparisons. DISCUSSION/SIGNIFICANCE OF IMPACT: Precision interventions to quit smoking are feasible for community smokers, who engaged at high rates. However, only 40% of providers supported patients’ quit attempts with medication prescriptions. Future research should test strategies to raise provider engagement in precision smoking treatment. CONFLICT OF INTEREST DESCRIPTION: R.F.T. has consulted for Quinn Emmanual and Apotex on unrelated topics. H.A.T. reported providing input on design for a phase 3 trial of cytisine proposed by Achieve Life Sciences and being a principal investigator of National Institutes of Health–sponsored studies for smoking cessation that include medications donated by the manufacturers. Other authors declare no potential conflicts of interest.

